# CircCCDC66: Emerging roles and potential clinical values in malignant tumors

**DOI:** 10.3389/fonc.2022.1061007

**Published:** 2023-01-09

**Authors:** Xiaoxiao Wang, Chao Zhang, Huangqin Song, Junlong Yuan, Lei Zhang, Jiefeng He

**Affiliations:** ^1^ Third Hospital of Shanxi Medical University, Shanxi Bethune Hospital, Shanxi Academy of Medical Sciences, Tongji Shanxi Hospital, Taiyuan, China; ^2^ Department of Hepatobiliary Surgery, Shanxi Bethune Hospital, Shanxi Academy of Medical Sciences, Tongji Shanxi Hospital, Taiyuan, China; ^3^ Hepatic Surgery Center, Institute of Hepato-Pancreato-Biliary Surgery, Tongji Hospital, Tongji Medical College, Huazhong University of Science and Technology, Wuhan, China

**Keywords:** circular RNA, circCCDC66, malignant tumors, endogenous competitive RNA, regulatory mechanism, cancer progression

## Abstract

Circular RNAs (circRNAs) are endogenous non-coding RNAs (ncRNAs) with a closed-loop structure. In recent years, circRNAs have become the focus of much research into RNA. CircCCDC66 has been identified as a novel oncogenic circRNA and is up-regulated in a variety of malignant tumors including thyroid cancer, non-small cell carcinoma, gastric cancer, colorectal cancer, renal cancer, cervical cancer, glioma, and osteosarcoma. It mediates cancer progression by regulating epigenetic modifications, variable splicing, transcription, and protein translation. The oncogenicity of circCCDC66 suppresses or promotes the expression of related genes mainly through direct or indirect pathways. This finding suggests that circCCDC66 is a biomarker for cancer diagnosis, prognosis assessment and treatment. However, there is no review on the relationship between circCCDC66 and cancers. Thus, the expression, biological functions, and regulatory mechanisms of circCCDC66 in malignant tumor and non-tumor diseases are summarized. The clinical value and prognostic significance of circCCDC66 are also evaluated, which can provide insights helpful to those exploring new strategies for the early diagnosis and targeted treatment of malignancies.

## Introduction

Malignant tumors remain diseases that threaten human life and health around the world. They are also one of the leading causes of human death (1, 2). Due to the novel coronavirus 2019 (COVID-19) pandemic, there has been a negative impact on the diagnosis and treatment of malignant tumors (3). The number of diagnosed cancer cases has reportedly decreased dramatically worldwide, with a significant increase in diagnosed cases expected in the near future (4). Therefore, there is an urgent need to identify potential highly specific biomarkers to improve the early diagnosis of cancer and enhance survival outcomes.

For many years, a great number of studies in tumor biology focused on the involvement of protein-coding genes, which accounted for less than 2% of the entire genome (5). With the development of whole genome sequencing, scientists have gained a better understanding of the transcriptome of organisms (6). More than 90% of the human genome contains non-coding RNAs (ncRNAs) that play an important role in biology, but ncRNAs were previously considered to be “transcriptional noise” or “transcriptional waste” (7). Circular RNAs (circRNAs) are a class of functionally diverse regulatory ncRNAs that are mainly produced by the circularization of exons or introns of precursor RNAs (pre-mRNAs) through alternative splicing followed by processing (8, 9). They are then reverse-spliced to form covalent closed-loop structures without a 5’ cap or 3’ Poly-A tail. In recent years, circRNAs have attracted much interest from scientists and there is a body of research evidence to the effect that circRNAs play an oncogenic or tumor suppressor role in the occurrence and development of cancers (10–12). For example, hsa_circ_0001666 inhibited epithelial-mesenchymal transition (EMT) and stemness of colorectal cancer (CRC) cells by competitively binding to miR-576-5p and high expression of hsa_circ_0001666 were associated with better clinical prognosis in CRC patients (13); CircZEB1 promoted the expression of PIK3CA by targeting miR-199a-3p to affect the proliferation and apoptosis of hepatocellular carcinoma (HCC) (14). This suggested that circZEB1 can be used as a biomarker for the diagnosis and treatment of HCC.

CircCCDC66 is a rising star in the circRNA family. CircCCDC66, also known as circ_0001313, is the full name of the circular RNA coiled-coil domain containing 66, which is the circular transcript from the CCDC66 pre-mRNA. CircCCDC66 was first reported to be up-regulated in CRC in 2017 (15). It was found to be closely related to the pathogenesis and prognosis of various human diseases (16–18). It not only participates in the regulation of the cell cycle (19), energy metabolism (20) and tumorigenesis through the network of competing endogenous RNAs (ceRNAs) (21, 22) but also regulates the expression of host proteins to exert a cis-transcriptional regulatory role (23). Due to its genome-wide expression patterns and tissue-specific expression signatures in various tissues, circCCDC66 has a strong potential as a novel biomarker and therapeutic target for cancers.

Here, we present a review of the latest progress in the biological functions and regulatory mechanisms of circCCDC66 in various malignant tumors. Furthermore, the role and significance of circCCDC66 in cancers are also evaluated and the prospects for future research are discussed.

## The characterization and function of circRNAs

CircRNAs are widely distributed in the cytoplasm and nucleus of eukaryotic cells, where they are abundantly, robustly, and conserved expressed (24) Compared with linear RNAs, circRNAs are resistant to RNAases, causing circRNAs to be conserved across species and having higher stability and longer half-life (25).Moreover, circRNAs account for a considerable proportion of transcripts, and some are even expressed in significantly higher abundance than others (26). The expression of circRNAs can be tightly regulated by RNA binding proteins (RBPs), and the co-localization of circRNAs and RBPs suggests that circRNAs may also affect RBP activity (27, 28).

**Figure 1:**
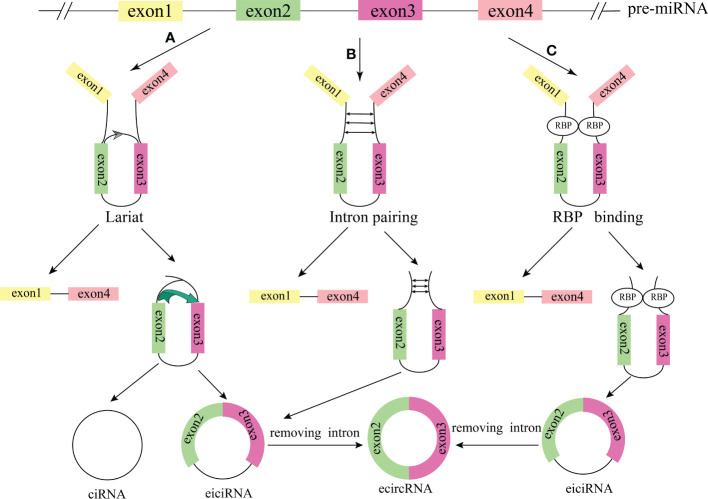
The formation process of the circRNAs. **(A)** Cyclization driven by lariat. **(B)** Intron pairing drives cyclization. **(C)** RBP was combined with cyclization to form a bridge. The pre-mRNA was spliced to produce an mRNA composed of exon 1 and exon 4 and an RBP binding RNA containing exons 2 and 3, and then generated ciRNA and eiciRNA. EiciRNA removed intron to form ecircRNA.

According to the sequence composition, circRNAs can be classified into three types (1): exonic circRNAs (ecircRNAs). EcircRNAs are exon-derived circRNAs consisting of single or multiple exons of a gene, and account for the majority of circRNAs (2); exon-intron circRNAs (eiciRNAs). EiciRNAs contain both exons and introns (3); intronic circRNAs (ciRNAs) (**Figure 1**). CiRNAs mainly derived from the lariat RNA and tRNA introns generated by pre-mRNA splicing. Another study reported that viral RNA genomes, rRNA, tRNA, and snRNA can also be circularized to generate circRNA (29). Recent studies have identified important regulatory functions of circRNAs. Cytoplasmic circRNAs (ecircRNAs) are mostly produced from exons and regulate gene expression at the post-transcriptional and translational levels, including interacting with different proteins in the cytoplasm, competing with microRNAs (miRNAs) to regulate mRNA metabolism, and encoding specific proteins or generating pseudogenes (30). The circRNAs (eiciRNA and ciRNA) distributed in the nucleus mainly regulate gene expression at the epigenetic and transcriptional levels, including interaction with proteins in the nucleus, regulation of parental gene expression, regulation of chromatin remodelling and processes of splicing and transcription (31, 32). Moreover, circRNAs can also be packaged in extracellular vesicles to form exosomes and transported in the circulation (33).

Accumulating evidence suggests that circRNAs are involved in the regulation of various tumor biological processes, especially playing a large regulatory role in important biological processes such as tumor proliferation, apoptosis, migration, invasion, chromatin remodeling, metabolism, and immune escape (32, 34). CircRPN2 accelerated ENO1 degradation and promoted glycolytic reprogramming through the AKT/mTOR pathway, thereby inhibiting HCC aerobic glycolysis and metastasis (35); plasma exosomal circLPAR1 specifically bound to eIF3h and inhibited the METTL3-eIF3h interaction, which reduced the translation of the oncogene BRD4 and inhibited CRC growth (36). These studies implied that circRNAs are critical for tumorigenesis and progression. Further exploration of the role of circRNAs in human cancer and their regulatory mechanisms could provide insights for exploring new strategies for early diagnosis and targeted therapy of cancers.

## The source of the circCCDC66-CCDC66

**Figure 2:**
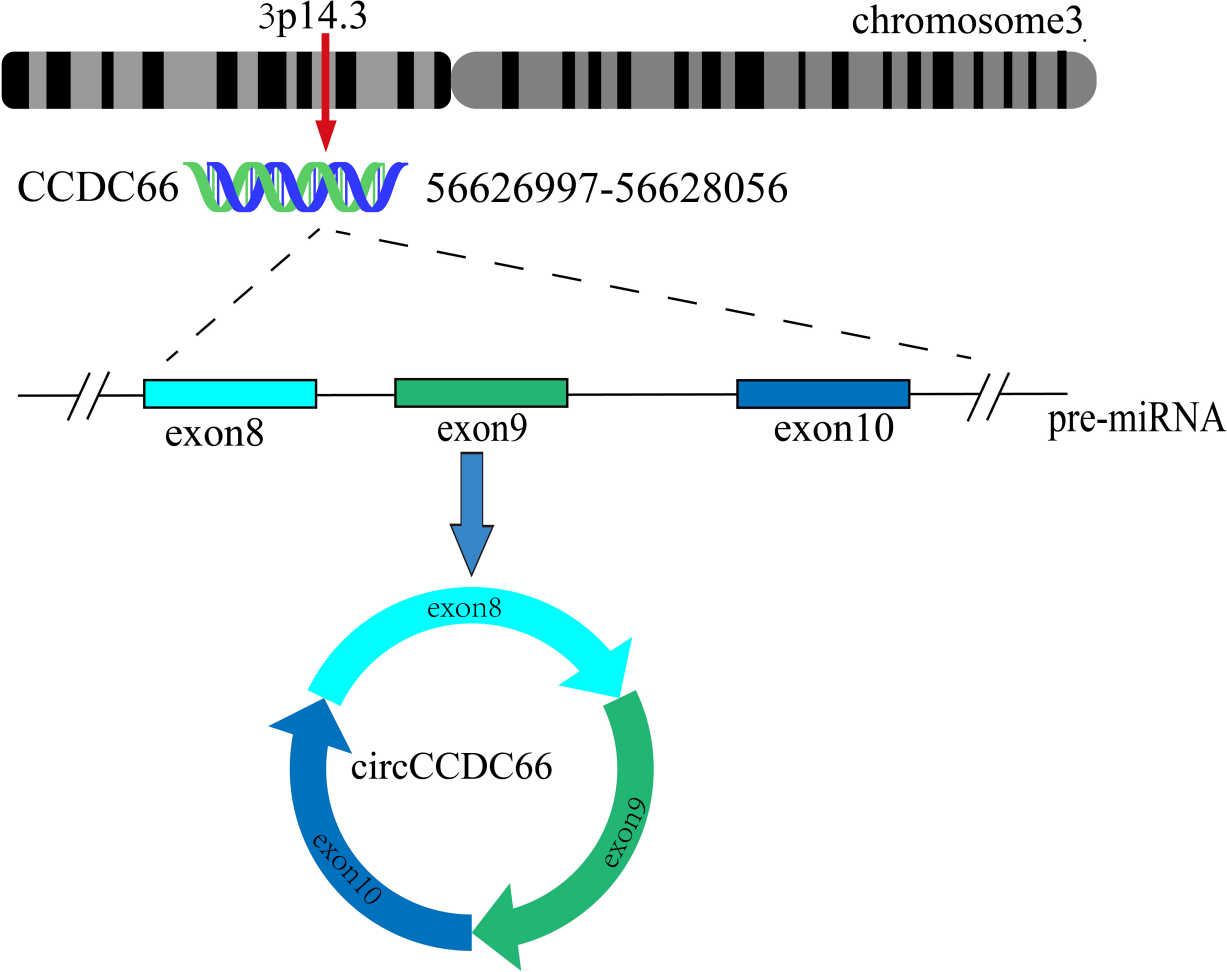
Schematic representation of the formation of circCCDC66. Exons 8-10 of CCDC66 located on chromosome 3 produce a 468-nucleotide circCCDC66.

CircCCDC66 was derived from exons 8-10 of the CCDC66 gene, located at chr3: 56626997-56628056. It has a full length of 468 nucleotides and is formed by non-linear splicing of CCDC66 pre-mRNA (15, 19) (**Figure 2**). According to the circRANA database (circBank and circBase), the circCCDC66 transcript was identified as hsa_circ_0001313 and showed high levels of conservation of among different species. Moreover, circCCDC66 is widely present in eukaryotic cells and is preferentially expressed in the cytoplasm.

The coiled-coil domain containing 66 (CCDC66) is a protein-coding gene located on chromosome 3p14.3. It was originally described as a gene mutated in retinal degeneration (37). Gerding et al. (38) used a CCDC66 mutant mouse model to reveal that CCDC66 deletion led to retinal degeneration and dysfunction. Subsequently, studies revealed that CCDC66 as a component of the centrosome and ciliary complex, co-localized with centriole satellites, microtubules, and molecular motors (39, 40). It also acts synergistically in ciliary formation, satellite organization, and ciliary recruitment in Bardet–Biedl syndrome4. Notably, retinal degeneration mutations disrupt the centrosome and ciliary protein localization and interaction of CCDC66. Moreover, Latour et al. (41) also found that CCDC66 was part of the Joubert syndrome (JBTS)-related protein module that maintains ciliary stability. In addition to ciliary function, CCDC66 could also control mitotic progression and cytokinesis by promoting microtubule nucleation and organization. Dysregulation of CCDC66 may contribute to carcinogenesis and ciliopathies (42).

## CircCCDC66 expression in malignant tumors and its regulatory mechanism

Current research has confirmed that circCCDC66 is abnormally up-regulated in various types of cancers including thyroid cancer, non-small cell cancer, gastric cancer, colorectal cancer, renal cancer, cervical cancer, glioma, and osteosarcoma. In particular, circCCDC66 plays a key role in important biological processes such as tumor proliferation, migration, invasion, cell cycle and apoptosis, chemotherapy resistance, radiosensitivity, and immune response (**Table 1**). Next, the mechanism of circCCDC66 expression in cancers and regulation of tumor progression are reviewed and summarized and the clinical potential of circCCDC66 as a prognostic biomarker and cancer therapeutic agent are further expounded. The results will contribute to the development of more effective cancer treatment strategies.

**Table 1:** Expression regulation and roles of circCCDC66 in various diseases including malignant tumors.

Disease types	Cell lines	Expression	Related miRNAs and proteins	Functional roles	Referances
Thyroid cancer	Nthy-ori 3-1, CAL62, TPC1	Up-regulation	miR-211-5p, PDK4	Promoting cell proliferation, migration, invasion and glycolysis	(20)
	Nttry-ori-3-1, IHH4, BCPAP, K1, TPC-1	Up-regulation	miR-129-5p, LARP1	Promoting cell proliferation, migration, invasion and tumor growth	(16)
Non-small cell lung carcinoma	PC-9, SPC-A1, A549, H1299, HBE	Up-regulation	STAT3, CCDC66, miR-33a-5p, KPNA4	Promoting cell proliferation, migration, invasion and tumorigenesis	(21)
	H1299, A549	Up-regulation	miR-211, SRCIN1	Promoting cell proliferation	(17)
Lung adenocarcinoma cell	H125, H23, H226, H838, H1437, H2009, H2087, A549	Up-regulation	HGF/c-Met, FAK, nAchR7α	Increasing EMT and drug resistance	(43)
Gastric cancer	BGC-823, MGC-803, SGC-7901, AGS, GES1	Up-regulation	miR-1238-3p, LHX2	Promoting cell proliferation, migration and invasion; induced cell cycle and suppressing cell apoptosis	(19)
	BGC-823, MGC-803, SGC-7901, HGC-27, GES-1	Up-regulation	E-cadherin, N-cadherin, Vimentin, Slug, c-Myc, TGF-β, p-SMAD2, MMP9	Promoting cell proliferation, migration and invasion, and suppressing cell apoptosis	(44)
	SGC7901, BGC 823	Up-regulation	miR-618, BCL2	Inhibiting cell apoptosis and promoting cisplatin resistance	(18)
Colorectal cancer	CCD 841 CoN, Caco-2, HCT116, HT-29, LS123	Up-regulation	/	Promoting cell proliferation, migration and metastasis	(15)
	SW480, SW620	Up-regulation	miR-338-3p	Promoting cell viability, colony formation, and caspase-3 activity, and reducing radio-sensitivity	(22)
	HCT116, HT-29	Up-regulation	PI3KK, DHX9	Promoting cell cycle and oxaliplatin-resistance, and suppressing cell apoptosis	(45)
	HCT116, SW620, NCM460, 293T	Up-regulation	miR‐3140, Beclin1, p62	Enhancing cell viability, migration, invasion and autophagy, and reducing the apoptosis of hypoxia-exposed cells	(46)
	RKO, HCT-116, SW620, SW480	Up-regulation	miR-370, MDM4	Promoting cell proliferation, migration and invasion	(47)
Renal cell carcinoma	Caki-1, Caki-2, 786-O, 767P, HK2, OS-RC-2, SN12C, SKRC39, ACHN, A498	Up-regulation	HGF/c-Met	Enhancing the cancer stem cells enrichment	(48)
Cervical cancer	C33A, HT-3, Hela, SiHa, H8, HEK-293T	Up-regulation	miR-452-5p, REXO1	Promoting cell proliferation, migration, invasion and tumorigenesis	(49)
Glioblastoma	HEB, U87, SW1783, U373	Up-regulation	miR-320a, FOXM1	Promoting cell proliferation, migration and invasion	(50)
Osteosarcoma	SW1353, U2OS	Up-regulation	miR-338-3p, PTP1B	Promoting cell proliferation, migration and metastasis	(51)
Hirschsprung’s disease	293T, SH‐SY5Y	Down-regulation	miR-488-3p, DCX	Inhibiting cell proliferation and migration	(52)
Osteoarthritis	HC-A	Up-regulation	miR-3622b-5p, SIRT3, IL-6, TNF-α	Inhibiting proliferation and promoting apoptosis of chondrocytes	(53)
Abdominal aortic aneurysm	VSMC	Up-regulation	miR-342-3p, CCDC66	Inducing proliferation facilitation and apoptosis reduction	(23)

## CircCCDC66 and thyroid cancer

Thyroid cancer (TC) is the most frequent endocrine malignancy worldwide and its incidence has steadily increased over several decades. Papillary thyroid cancer is the most common TC histotype, accounting for approximately 85% of all TCs (54). Differentiated TC behaviors vary from indolent to extremely aggressive, and TC prognosis is largely based on clinicopathological features that are a direct consequence of altered cellular and tissue microenvironment (55). Therefore, there is an urgent need for an in-depth understanding of the molecular mechanisms of TC invasion and metastasis for better assessment of invasion risk and clinical guidance.

CircRNAs play critical roles in gene regulation at the epigenetic, transcriptional, and post-transcriptional levels. Ren et al. (20) found that the level of expression of circCCDC66 was significantly up-regulated in TC tissues compared with normal thyroid tissues (n=9, P<0.05). CircCCDC66 might promote TC progression. *In vitro*, knockdown of circCCDC66 resulted in decreased expression of targeted miR-211-5p, which inhibited TC cell proliferation, migration, invasion, and glycolysis. miR-211-5p has been reported to be involved in the metabolic activity of various cancer cells (56). Overexpression of miR-211-5p significantly reduced the expression of pyruvate dehydrogenase kinase 4 (PDK4) and circCCDC66. Inhibition of PDK4 by miR-211 induced oxidative phosphorylation (20). These may reduce glucose and increase the expression of pyruvate dehydrogenase and key enzymes in the tricarboxylic acid cycle, which can affect the process of glucose metabolism and glucose homeostasis. Thus, circCCDC66 may upregulate the expression of PDK4 by binding to miR-211-5p by sponging, thereby the progression of TC.

Subsequently, Li et al. (16) also found that the level of expression of circCCDC66 was significantly increased in papillary TC (PTC) tissues (n=60, P<0.05) and cell lines. This study also investigated the relationship between the level of expression of circCCDC66 and clinicopathological features. The level of expression of circCCDC66 was positively correlated with tumor size (P=0.0099), TNM stage (P=0.0362), and lymph node metastasis (P=0.0089) in patients with PTC (16). Further mechanistic studies showed that circCCDC66 could bind miR-129-5p to promote the expression of La-related protein 1 (LARP1), which may accelerate the malignant progression of PTC. Therefore, targeting circCCDC66 may be considered a promising TC therapeutic strategy.

## CircCCDC66 and lung cancer

Lung cancer (LC) is the most common cancer and the leading cause of cancer death worldwide. There are estimated 2.09 million new cases and 1.76 million deaths globally each year (57). According to histological type, lung cancer is divided into small-cell lung carcinoma (SCLC) and non-small cell lung carcinoma (NSCLC). NSCLC is the most common and deadliest type of primary lung cancer, accounting for 85% of all lung cancers. NSCLC includes lung adenocarcinoma (LADC), lung squamous cell carcinoma (LUSC), and large cell lung carcinoma (LCLC). These are usually diagnosed at a later stage. Despite significant advances in NSCLC surgery, chemotherapy, and targeted therapy over the past two decades, due to the difficulty of early diagnosis, easy metastasis, and poor prognosis, the current five-year survival rate of NSCLC remains below 20% (58). Therefore, it is necessary to reveal the occurrence and molecular mechanisms of lung cancer to find new and reliable biomarkers and therapeutic targets.

In recent times, a better understanding of disease biology and the identification of oncogenic driver alterations have altered the therapeutic landscape. Emerging evidence suggests that circRNAs play an important role in the initiation and progression of non-small cell lung cancer, with the potential to play a role in the development and progression of non-small cell lung cancer. Research has been undertaken to examine potential cancer biomarkers (59). Wang et al. (21) found that the expression level of circCCDC66 was up-regulated in NSCLC cells. Knockout of circCCDC66 could inhibit the proliferation, migration, and invasion of NSCLC cells while promoting the apoptosis of NSCLC cells. CircCCDC66 acts as a sponge of miR-33a-5p to mediate the ceRNA mechanism to regulate gene expression post-transcriptionally. Existing evidence proves the tumor suppressor role of miR-33a-5p in a variety of cancers (60). Karyopherin subunit alpha 4 (KPNA4) has been shown to be an mRNA with the potential to promote tumorigenesis and the development of various human malignancies (61). As revealed by the study, up-regulation of circCCDC66 accelerates the progression of NSCLC by binding miR-33a-5p to up-regulate KPNA4 expression. In addition, signal transducer and activator of transcription 3 (STAT3) was confirmed to activate CCDC66 transcription and increase the expression of circCCDC66 in non-small cell lung cancer cells. This provides evidence for the oncogenic role of circCCDC66 in NSCLC cells (21). Subsequently, Hong et al. (17) found that circCCDC66 can also bind miR-211 by acting as a competitive adsorbent for ceRNA, causing a significant increase in the expression of SRC kinase signaling inhibitor 1 (SRCIN1). This provides a new perspective on the post-transcriptional regulatory mechanism of circCCDC66. It is expected to be a prospective molecular biomarker for detection and treatment of NSCLC.

LADC is the most common histological subtype of non-small cell lung cancer. The morbidity and mortality of LADC are increasing year-on-year due to early tumor recurrence, enhanced resistance to chemoradiotherapy, and rapid progression of the disease (62). Chemotherapy targeting mutated tyrosine kinase inhibitors (TKIs) of the epidermal growth factor receptor (EGFR) has recently become the main strategic treatment for LADC patients, such as in gefitinib, erlotinib, and afatinib (63). Joseph et al. (43) discovered that SUMO-activating enzyme subunit 1 (SAE1) and circCCDC66 were highly expressed in LADC. Hepatocyte growth factor (HGF) and HGF receptor (or c-Met) positively regulates the expression of SAE2 and circCCDC66 to induce EGFR resistance and increase the EMT and metastatic potential of LADC cells. This finding is consistent with the mechanism of action reported in CRC (48). Designing multimodal drugs targeting multiple targets in EGFR and resistance-related feedback loops in the future may provide additional benefits to cancer patients.

## CircCCDC66 and gastric cancer

Gastric cancer (GC) is one of the most common highly aggressive malignancies worldwide and the second leading cause of cancer-related death. GC remains a global health problem. The survival rate of GC patients has improved significantly over the past few decades (64). However, due to the lack of specific symptoms in the early stage of GC, most gastric cancer patients are often diagnosed at an advanced stage, and it is prone to peritoneal, local, and distant metastases. GC has a high recurrence rate and its prognosis remains suboptimal. The current 5-year survival rate for advanced gastric cancer is less than 20%. If timely and accurate diagnosis and comprehensive treatment are performed before the tumor invades the basement membrane, the 5-year survival rate of GC patients may be as high as 90% (65). Therefore, early detection with effective screening methods is critical to improve the long-term survival of patients.

Gastric cancer is a highly molecularly and phenotypically heterogeneous disease. Recent advances in the genomic analysis indicate that highly-expressed circCCDC66 enhances the malignant phenotype of tumor cells and is closely associated with poor prognosis (5, 18, 19). Yang et al. (60)19 found that circCCDC66 may promote cell proliferation and invasion by binding miRNA-1238-3p to up-regulate the expression of LIM-homeobox domain 2 (LHX2) to accelerate the progression of GC. LHX2 has been shown to be a member of the LIM homeodomain protein family involved in the formation and development of various tumor cells (66). Knockout of circCCDC66 can significantly inhibit the proliferation and invasion of GC cells, block cell cycle progression in G0/G1 phase and induce GC cell apoptosis. Overexpression of miRNA-1238-3p or silencing of LHX2 reversed the promotional effect of circCCDC66 on GC cell migration and invasion (19). This is the first report on the pattern of expression and biological function of circCCDC66 in gastric cancer. The circCCDC66/miRNA-1238-3p/LHX2 axis may be a promising target for GC therapy, providing new evidence that circRNAs function as miRNA sponges.

In addition, Xu et al. (44) also confirmed that circCCDC66 was up-regulated in GC tissues (n=70, P<0.001) and cell lines. Its level of expression is correlated with tumor stage (P=0.01) and lymphatic metastasis (P=0.014). Knockdown of circCCDC66 inhibited the activation of c-Myc and TGF-β signaling pathways. Both published reports and experimental evidence support c-Myc as a powerful oncogene (67). Overexpression of c-Myc can increase the ability of cancer cells to metastasise by directly or indirectly modulating the ability of EMT molecular markers to induce cell motility. TGF-β regulates tumorigenesis, progression, and metastasis through extensive and complex interdependent interactions (68). circCCDC66 induces EMT and promotes GC growth and metastasis by activating c-Myc and TGF-β signaling pathways. This suggests that it may serve as a potential biomarker for GC.

Cisplatin (CDDP) is a first-line chemotherapy drug for gastric cancer, which induces tumor cell apoptosis by interfering with DNA replication. Subsequently, Zhang et al. (18)proposed in a recent study that circCCDC66 is an important regulator of the development of CDDP resistance. Studies have shown that circCCDC66 significantly inhibits B-cell lymphoma-2 (BCL2) mRNA and protein levels by targeting miR-618 to induce cisplatin chemotherapy resistance in gastric cancer cells. For CDDP-sensitive cells, CDDP resistance resulted in marked up-regulation of BCL2. BCL2 was confirmed to be a key oncogene that regulates apoptosis signaling pathways in tumors (69). Overexpression of BCL2 can reverse the CDDP resistance of circCCDC66-suppressed GC cells by knocking down circCCDC66, which may provide a new theoretical basis for the treatment of GC.

## CircCCDC66 and colorectal cancer

CRC is one of the most common and aggressive human malignancies and the third leading cause of cancer-related death worldwide. According to reports, the global burden of the disease will be increased by 60% by 2030, with more than 2.2 million new cases and 1.1 million deaths (70). Despite major advances in prevention and treatment, the 5-year survival rate for colon cancer patients remains at 30% due to recurrence and metastasis (71). Therefore, there is an increasing need to discover promising biomarkers for CRC diagnosis.

More studies have shown that abnormally expressed circRNAs are involved in the occurrence and development of CRC by acting as competing endogenous RNAs (72). CircCCDC66 is a recently discovered novel circRNA that is up-regulated in colon cancer tissues and promotes colon cancer progression. Hsiao et al. (15) performed RNA-Seq analysis on 12 pairs of CRCs and paired non-tumor samples to identify 74 novel circRNAs clinically relevant to CRC. They have further been validated by quantitative reverse transcription polymerase chain reaction (qRT-PCR). Among them, the expression of circCCDC66 was up-regulated in CRC cell lines and clinical specimens, which was negatively correlated with prognosis (n=12, P<0.05). *In vitro* and *in vivo* experimental studies showed that circCCDC66 regulates colorectal oncogenes with multiple tumor characteristics including cell proliferation, migration, invasion, and anchorage-independent growth. CircRNAs contain different binding sites for different miRNAs and have oncogenic capabilities by sponging to protect multiple oncogenes from miRNA attack (73). This is the first report on the regulatory role and clinical significance of circRNA CCDC66 in human cancer progression. It also highlights the importance of complex ceRNA-regulated gene networks in cancer progression and therapeutic efficacy.

Subsequently, Wang L. et al. (22) found that the expression of circCCDC66 in radioresistant colon cancer tissues was significantly higher than that in radiosensitive tissues and that circCCDC66 was significantly up-regulated in colon cancer cells exposed to radiation (n=40, P<0.001). Knockdown of circCCDC66 reduced cell viability, inhibited colony formation rate, and increased caspase-3 activity in irradiated colon cancer cells. While the expression of miR-338-3p was down-regulated, miR-338-3p could reverse the effects of circCCDC66 on cell viability, colony formation, and caspase-3 activity. miR-338-3p as considered a tumor suppressor is frequently found to be reduced in various cancer types. Previous studies have reported an important role for miR-338-3p in cancer chemotherapy resistance (74). This finding revealed the biological function and mechanism of circCCDC66 in the radiosensitivity of CRC. CircCCDC66 regulates the radioresistance of colon cancer by negatively regulating miR-338-3p expression, which may provide a potential therapeutic target for radioresistant colon cancer patients.

CircRNAs can be derived not only from cancer cells, but also from cells in the tumor microenvironment, immune cells, or cells of other organs (75). CircRNAs can be selectively secreted into the blood, and their expression in plasma differs from that in tissues. This is a phenomenon related to its active secretion mechanism and function. Recently, plasma circRNAs have been shown to act as biomarkers for CRC. Lin et al. (76) found that the expression levels of circCCDC66, circABCC1, and circSTIL were decreased in the plasma of CRC patients compared with healthy controls by the training cohort (n=15 *vs*.15) and the validation cohort (n=30 *vs*. 46) of CRC. The sensitivity and specificity of the three circRNA groups (circCCDC66 AUC=0.756 P<0.001; circABCC1 AUC=0.663 P=0.0041; circSTIL AUC=0.677 P=0.0018) were also evaluated together and had some diagnostic value. Notably, the combination of three circRNAs and the traditional serum tumor protein biomarkers CEA and CA19-9 will improve the rate of detection of early CRC and reduce the missed diagnosis rate (AUC=0.885, P=0.0021). High-throughput methods such as microarrays and next-generation sequencing should be used in the future to characterize the global landscape of plasma circRNAs in a cohort of human subjects. This may identify the most valuable diagnostic and prognostic factors for CRC.

The formation of CRC is a complex and dynamic process. Treatment failure in CRC is often associated with recurrent and drug-resistant cancer tissue. Lin et al. (45) found that the expression of circCCDC66 was up-regulated in oxaliplatin-resistant CRC cells. The induction of circCCDC66 is dependent on oxaliplatin-induced DExH-box helicase 9 (DHX9) phosphorylation, which is required for the establishment of chemoresistance to oxaliplatin. According to related reports, DHX9 has a phosphorylation site for the DNA-dependent protein kinase phosphatidylinositol 3-kinase-related kinases (PI3KKs). DHX9 is phosphorylated near the substrate-binding domain mediated by PI3KK upon DNA damage (77). Studies have shown that blocking PI3KK activity or DHX9 phosphorylation by point mutation reduces oxaliplatin-induced circCCDC66 expression and the ability to generate chemoresistant cells. This study proposes that DHX9 phosphorylation, which is impaired by cytotoxic stress, regulates chemotherapy-resistant CRC by promoting oncogenic circRNA expression, thereby providing mechanistic insights for the development of therapeutic strategies for recurrent and chemoresistant CRC.

Considering that intra-tumoral hypoxia is one of the driving forces of tumorigenesis and development, Feng et al. (46) demonstrated for the first time that hypoxia-induced up-regulation of circCCDC66. CircCCDC66 knockdown decreased the proliferation, migration, and invasion of CRC cells and increased apoptosis when exposed to hypoxic conditions. It has been reported that miR-3140 functions as an oncogene or tumor suppressor both in vitro and in vivo (78). Research shows that circCCDC66 activates the autophagy pathway by sponging and binds miR-3140 to promote the malignant phenotype of CRC, which provides a new therapeutic strategy for CRC. There is increasing evidence that the mouse double minute 4 (MDM4) and mouse double minute 2 (MDM2) oncoproteins are key negative regulators of the p53 tumor suppressor (79). In addition, Mo et al. (47) showed that circCCDC66 up-regulated the expression of MDM4 by targeting miR-370 to promote cell proliferation, migration, and invasion in CRC. In conclusion, circCCDC66 acts as an oncogenic circRNA in CRC tumor development. These studies revealed the potential functions of circRNAs in the occurrence and development of CRC, which can provide new insights into the treatment and prevention of CRC.

## CircCCDC66 and renal cancer

Renal cancer is one of the most common and aggressive malignant tumors of the urinary system, accounting for more than 90% of all renal malignancies. Obesity and smoking are significantly associated with the pathogenesis of several systemic cancers, including renal cell carcinoma (RCC) (80). Changes in epigenetic phenomena, such as DNA methylation levels, and expression of lncRNAs and circRNAs, were observed early in the development of cancer (81). A deeper understanding of the pathophysiology of RCC may reveal relevant molecules able to advance the therapeutic management thereof.

A key focus of attention is the effect of circRNAs on renal cancer stem cells. Yang et al. (48) found that circCCDC66 was up-regulated not only in renal cancer cell lines, but also in cancer stem cell spheroids. More importantly, the results showed that circCCDC66 enhanced the enrichment of cancer stem cells. Further mechanistic studies indicated that the hepatocyte growth factor (HGF)/c-Met pathway was activated in cancer stem cell enrichment. The HGF/c-Met pathway could lead to up-regulation of circCCDC66. Inhibition of HGF/c-Met blocked circCCDC66-induced enrichment of cancer stem cells. According to related reports, HGF/c-Met positively regulates circCCDC66 to increase the EMT and metastatic potential of lung adenocarcinoma cells (43). If the combination of c-Met inhibitor and circCCDC66 inhibitor blocked cancer stem cell enrichment, it could provide new insights into cancer stem cells. Thus, circCCDC66 may be a promising biomarker and therapeutic target for renal cancer therapy.

## CircCCDC66 and cervical cancer

Cervical cancer remains the fourth most common cancer in women worldwide. Almost all cases of cervical cancer are caused by human papillomavirus infection (82). Persistent infection with human papillomavirus can lead to cervical intraepithelial neoplasia or precancerous lesions of adenocarcinoma, evolving *in situ* into cervical cancer. Therefore, it is necessary and indeed pressing to detect biomarkers for early diagnosis and prognosis.

Recent studies have reported that circRNAs are abnormally expressed in cervical cancer and are important regulators of cervical cancer carcinogenesis and progression (83). Zhang et al. (49) found that circCCDC66 was highly expressed in cervical cancer tissues (n=36, P<0.01). Knockdown of circCCDC66 attenuated the proliferation, migration, and invasion of cervical cancer cells *in vitro* and inhibited cervical cancer tumor growth *in vivo*. This was correlated with advanced tumor stage and larger tumor size. The association between circCCDC66 and miR-452-5p or RNA exonuclease 1 homolog (REXO1) was assessed by way of bioinformatics analysis, biotinylated RNA pull-down, RNA immunoprecipitation, and dual-luciferase reporter assay analysis. miR-452-5p has been shown to be involved in the progression of a variety of cancers including renal carcinoma (84), hepatocellular carcinoma (85), and lung squamous cell carcinoma (86). It plays a specific role in tumorigenesis and progression. This study shows that circCCDC66 competitively binds miR-452-5p through spongeization, and up-regulates REXO1 expression to aggravate the progression of cervical cancer (49). This may provide new insights into new therapeutic targets for basic research and clinical management of cervical cancer.

## CircCCDC66 and glioblastoma

Glioblastoma (GBM) is the most common and malignant primary brain tumor in adults. Its incidence increases with age, peaking at an age of 80 years. GBM accounts for 12% to 15% of all intracranial tumors and the five-year survival rate is only 5% (87). It is primarily characterized by extensive intra-tumoral and inter-tumoral genetic and epigenetic variability.

In recent years, a greater understanding of disease biology and the identification of oncogenic driver alterations have altered the therapeutic landscape. Emerging evidence suggests that aberrantly expressed circRNAs play a key role in the occurrence and development of GBM (50). Qi et al. (88) revealed that circCCDC66 was down-regulated in high-grade gliomas, and knockdown of circCCDC66 inhibited the proliferation, migration, and invasion of glioma cells. Co-transfection of miR-320a or forkhead box M1 (FOXM1) plasmids into glioma cells could rescue the consequences of si-circCCDC66-induced cell growth inhibition to some extent. Importantly, FOXM1 was identified as a key target of circCCDC66 involved in regulating DNA damage response pathways (89). This highlights the fact that circCCDC66 is overexpressed in gliomas and acts as an oncogene. It exerts oncogenic effects by regulating the ceRNA network, thus providing some new experimental evidence for its use in the clinical treatment of glioma.

## CircCCDC66 and osteosarcoma

Osteosarcoma (OS) is deemed to be a tumor derived from osteogenic mesenchymal stem cells and is the most common primary bone malignancy. It primarily affects the metaphyseal region of long tubular bones (proximal tibia or humerus and distal femur) (90). The peak incidence of OS is mainly concentrated in children and adolescents, and its rate of growth is high. It also has the characteristics of strong invasiveness, strong resistance to chemotherapy, poor prognosis, and a high rate of recurrence (91).

The pathogenesis of OS is currently unclear, and it is important to identify new therapeutic targets to combat this devastating and potentially fatal disease. Recent reports indicate that circRNAs are abnormally expressed in OS and are important regulators of OS carcinogenesis and progression (51). Xiang et al. (92) found high expression of circCCDC66 in tissue samples (n=12, P<0.05) and cell lines derived from OS patients by qRT-PCR. Down-regulation of circCCDC66 enhanced the interaction between miR-338-3p and protein tyrosine phosphatase 1B (PTP1B) and inhibited OS cell proliferation and metastasis. PTP1B has been identified as an intracellular checkpoint that is up-regulated in tumor T cells (93). Further analysis showed that circCCDC66 up-regulated the expression of PTP1B by sponging miR-338-3p (92). It limits T cell expansion and cytotoxicity leading to the malignant phenotype of osteosarcoma. This suggests that the circCCDC66/miR-338-3p/PTP1B axis may be a potential therapeutic target. CircCCDC66 can be used as a novel biomarker for OS screening to predict poor prognosis and monitor cancer progression.

## CircCCDC66 expression in human non-tumor disease and its regulatory mechanism

### CircCCDC66 and Hirschsprung disease

Hirschsprung disease (HSCR) is a developmental disorder of the enteric nervous system. It is caused by defects in neural crest cell proliferation, migration, and differentiation during enteric nervous system formation (94). This neural crest disease affects 1 in 5000 births worldwide. It is 80% heritable and usually manifests quickly after birth (95). The pathogenesis of HSCR is a complex process that requires strict regulation.

There is increasing evidence that circRNAs have been shown to promote the growth and metastasis of malignant tumors by sponging and adsorbing miRNAs (28). This may also lead to the appearance of HSCR by affecting the basic functions of the cell. Wen et al. (52)found that circCCDC66 is widely present in enteric neurons, while circCCDC66 is absent in HSCR patient samples. Low expression of circCCDC66 in intestinal tissue can inhibit HSCR intestinal epithelial cell proliferation and migration. This result has important implications for the progression of HSCR. Mutations in doublecortin have been reported to cause defects in neuronal migration and proliferation (96). CircCCDC66 could regulate cell proliferation and migration in HSCR by attenuating doublecortin expression by sponging miR-488-3p. This is the first study to demonstrate a potential link between the pathogenesis of Hirschsprung’s disease and circCCDC66 and its associated RNAs and proteins. Therefore, this provides a new idea for the diagnosis of HSCR.

### CircCCDC66 and osteoarthritis

Osteoarthritis (OA) is the most common chronic degenerative joint disease, which is characterized by articular cartilage degeneration and secondary bone hyperplasia (97). More than 500 million people worldwide are currently affected by OA. It peaks at an age of 75 years and is associated with high morbidity and disability (98), however, the exact molecular mechanisms that regulate the pathogenesis of OA remain unclear.

Importantly, studies have highlighted that circRNAs regulate their proliferation, apoptosis, autophagy, inflammation, or extracellular matrix reorganization by interacting with components in the OA microenvironment, such as chondrocytes, synoviocytes, and macrophages (99). Zhang et al. (53) first found that circCCDC66 was up-regulated in OA cartilage cells. Overexpression of circCCDC66 increased IL-6 and TNF-α levels in chondrocytes. Sirtuin 3 (SIRT3) as one of the most prominent sirtuins in mitochondria has been shown to be involved in various aspects of mitochondrial metabolism and homeostasis (100). Further mechanistic analysis implied that circCCDC66 promoted OA chondrocyte apoptosis by regulating the miR-3622b-5p/SIRT3 axis. This finding revealed novel aspects of the cellular functions and pathophysiological roles of circCCDC66 and miR-3622b-5p. CircCCDC66 can be considered as a potential molecular target for OA therapy.

### CircCCDC66 and abdominal aortic aneurysm

Abdominal aortic aneurysm (AAA) is an asymptomatic and fatal cardiovascular disease characterized by localized dilation of the abdominal aortic vessel wall. Degradation of the aortic wall matrix and increased vascular smooth muscle cells (VSMCs) are important pathological features in the formation and exacerbation of AAA (101). Due to the lack of effective surgical methods and the unpredictability of the disease, the aortic wall is constantly enlarging. This leads to wall rupture and severe bleeding with a mortality rate of up to 80% (102).

Existing evidence suggests that circRNAs, as novel pathological regulators, may be involved in the pathogenesis of AAA (103). Yang et al. (23) found that circCCDC66 was significantly located in the VSMC cytoplasm in AAA. CircCCD66 was elevated in angiotensin II-stimulated VSMCs. Knockdown of circCCDC66 induced enhanced proliferation and decreased apoptosis in VSMCs. The study also showed that circCCDC66 positively regulated the expression of its host gene CCDC66. Moreover, miR-342-3p had the strongest binding affinity to circCCDC66 and CCDC66. Notably, miR-342-3p has been shown to be a potent tumor suppressor in non-small cell lung cancer (104), hepatocellular carcinoma (105), and glioma (106), among others. CCDC66 has only been reported in retinal degeneration and dysfunction (26). CircDC66 acts as a miR-34-3p sponge in AAA to regulate the CCDC66-dependent growth of VSMCs. The circCCDC66/miR-342-3p/CCDC66 axis was shown to play a role in regulating the proliferation and apoptosis of VSMCs. This provides us with a new molecular mechanism of action associated with AAA.

## The roles of circCCDC66 in cancers

**Figure 3:**
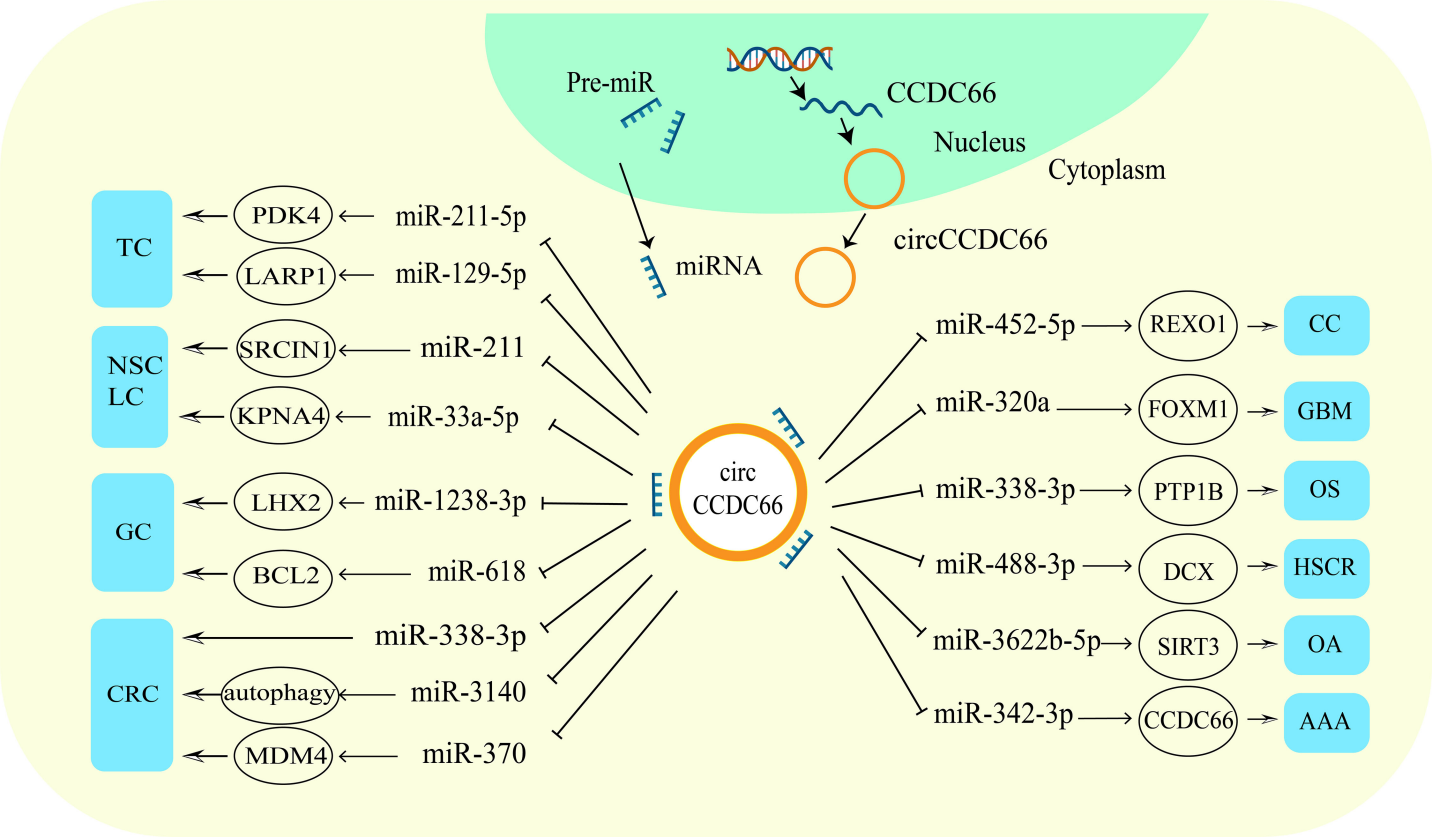
CircCCDC66 participates in various diseases through different ways. Up-regulation of circCCDC66 mediates the occurrence and development of malignant tumors.

As a member of the circRNAs family, circCCDC66 is aberrantly expressed in thyroid cancer, non-small cell cancer, gastric cancer, colorectal cancer, renal cancer, cervical cancer, glioma, osteosarcoma and other cancers. It affects cancer development by regulating tumor malignant behavior. Mechanistically, circCCDC66 functions as an oncogene in cancer by acting as a molecular sponge competitively adsorbing miRNAs and thus regulating the expression of target genes (**Figure 3**). The networks of circCCDC66-regulated miRNAs are a key component of oncogene activation. CircCCDC66 promoted PDK4 expression by repressing miR-211-5 and up-regulated LARP1 expression by binding to miR-129-5p to accelerate thyroid cancer progression (16, 20). In NSCLC, circCCDC66 promoted cell proliferation through miR-211/SRCIN1 axis (17). Moreover, circCCDC66 was highly expressed in precancerous polyps and cancer tissues of colon cancer, and exacerbated the proliferation, migration and invasion of CRC cells by competitively binding miR-370 to upregulate MDM4 expression (15, 47). And circCCDC66 could also exert oncogenic roles by binding miR-1238-3p and miR-452-5p in gastric and cervical cancers (19, 49).

On the other hand, circCCDC66 also acts as an miRNA sponge to regulate gene expression in non-neoplastic diseases. For example, circCCDC66 binding with miR-342-3p in abdominal aortic aneurysm up-regulated the host gene CCDC66 to induce cell proliferation and reduce apoptosis (23), while respectively binding with miR-488-3p and miR-3622b-5p suppressed the proliferation capacity of cells in Hirschsprung’s disease and osteoarthritis (52, 53). **Table 1** shows the miRNAs and target proteins regulated by circCCDC66 in a variety of cancers.

Cancer stem cells (CSCs) can regulate the growth of cancers due to their self-renewal ability and high tumorigenic activity (107). Moreover, it plays an important role in chemotherapy resistance and metastatic recurrence. Recent studies have revealed that circRNAs regulate key gene expression and signaling pathways associated with CSCs, which has attracted significant attention. For example, circFAT1 promoted cancer stemness and immune evasion by activating STAT3 (108); circIPO11 recruited TOP1 to the GLI1 promoter to trigger its transcription and to drive self-renewal of the liver CSCs (109). The current study of circCCDC66 in CSCs has only been reported in renal cancer stem cells. HGF/c-MET-induced up-regulation of circCCDC66 expression promoted enrichment of renal cancer stem cells and accelerated the malignant process of cancer (48). Combined with the results of CSCs diversity and plasticity studies, blocking the source of CSCs and fully characterizing them may provide a prerequisite for therapeutic cancer strategies targeting CSCs.

Intratumoral hypoxia is one of the driving forces of cancer progression, triggering a variety of cellular metabolic stresses and also leading to increased autophagic activity (110). Exposure to hypoxic conditions increased the expression of circCCDC66 in CRC. Hypoxia-induced circCCDC66 combined with miR-3140 to activate the pathway of autophagy, which leaded to CRC cell growth and metastasis (46). Overexpression of circCCDC66 also acted as an miRNA sponge to interfering with miR-338-3p to promote cell viability and colony formation, and reduced radiosensitivity (22).

In addition, circCCDC66 plays a key role in cancer chemoresistance. CircCCD66 expression was dependent on oxaliplatin induction, and blocking PI3KK activity or DHX9 phosphorylation by point mutation decreased circCCDC66 expression and the ability to generate chemoresistant cells (45). Meanwhile, circCCDC66 is an important regulator in the progression of CDDP resistance. CircCCDC66 was overexpressed in CDDP-resistant gastric cancer cells and tissues. It promoted the up-regulation of BCL2 to inhibit apoptosis by targeting binding to miR-618 (18). However, there are no targeted inhibitory drugs for circCCDC66. Future research is needed not only to further explore the specific mechanisms by which circCCDC66 regulates cancer, but also to develop drugs that target circCCDC66 to reduce the organism’s resistance to chemotherapeutic agents and enhance the sensitivity of tumors to radiotherapy.

## The clinical significance of circCCDC66 and its prognostic value

Human tumor specimens are used as the “gold standard”. Studies have found that circCCDC66 is up-regulated in a variety of solid tumors, including thyroid cancer, non-small cell cancer, gastric cancer, CRC, renal cancer, cervical cancer, glioma, osteosarcoma, *etc.* It is positively correlated with clinicopathological features such as tumor size, TNM stage, lymphatic metastasis, and vascular invasion. GC patients receiving cisplatin chemotherapy had decreased 5-year disease-free survival (DFS) in tissues with high expression of circCCC66 (n=53) compared to low expression of circCCC66 (n=52), which predicted a poorer prognosis (P=0.020) (18). Multivariate regression analysis showed that circCCD66 level was an independent risk factor for DFS (HR=1.775, P=0.011). Another study supported reduced plasma circCCDC66 levels in precursor lesions of CRC (colonic adenomas and adenomatous polyps), which may be a potential biomarker for CEA-negative or CA199-negative in CRC (76). Furthermore, circCCDC66 is sensitive to the state of tumor cells and may show the opposite state of expression when stimulated by apoptosis (**Table 2**).

**Table 2:** Clinical pathological features and prognostic significance of circCCDC66 in malignant tumors.

Cancer types	Samples	Clinicopathological features	Prognostic implication	Referances
Thyroid cancer	60 pairs of tissues and adjacent normal tissues	Tumor size (P=0.0099) TNM stage (P=0.0362) Lymph node metastasis (P=0.0089)	poor prognosis	(16)
Gastric cancer	50 pairs of tissues and adjacent normal tissues	Lower overall survival (P=0.0252)	poor prognosis	(17)
	70 pairs of tissues and adjacent normal tissues	Tumor stage (P=0.01) Lymphatic metastasis (P=0.014)	poor prognosis	(44)
	105 patients accepted cisplatin-based chemotherapy with GC	Histological grade (P=0.001) Clinical stage (P=0.022) T classification (P=0.016) Cisplatin chemosensitivity (P=0.009) An independent risk factor for disease-free survival (P=0.011)	poor prognosis	(18)
Colon cancer	76 nontumor tissues, 131 tumor tissues and 22 polyps	Lower overall survival (P<0.05)	poor prognosis	(15)
Cervical cancer	36 pairs of CC tissues and adjacent normal tissues	TNM stage (P<0.05) Tumor size (P<0.01)	poor prognosis	(49)

Notably, the combined detection of circCCDC66 and traditional tumor markers can improve the specificity and sensitivity of diagnosis. Thus, circCCDC66 plays an important role in the occurrence, development, and prognosis of various cancers. In the future, we will expand the exploration of multiple areas of this important molecule, which will help to develop more effective cancer treatment strategies.

## Conclusions and future expectations

As a new class of ncRNAs, thousands of circRNAs are important regulators of human gene expression. In recent years, circCCDC66 has been identified as a novel oncogenic circRNA with up-regulated expression in a variety of malignancies and significantly correlated with important clinical features such as tumor size, histological grade, TNM stage, and overall survival, among others. The current research on circCCDC66 mainly focuses on the molecular mechanism of action in cancers. To the best of our knowledge, circCCDC66 indirectly promotes oncogene expression by competitively binding to miRNAs (miR-211-5p/PDK4, miR-33a-5p/KPNA4, miR-320a/FOXM1, *etc*). At the same time, it activates the c-Myc/TGF-β signaling pathway and the autophagy pathway to regulate tumor cell proliferation, migration, invasion, drug resistance, autophagy, and EMT. In addition, circCCDC66 also plays an important regulatory role in non-tumor diseases such as Hirschsprung’s disease, osteoarthritis, and abdominal aortic aneurysm. These illustrate the heterogeneity of circRNA expression and the diversification of functions, however, research of the mechanism of action of circCCDC66 in cancers remains rare.

As an element in a dynamic network of ncRNA machinery, circCCDC66 has multiple functions in tumor progression. In the future, more detailed studies of the specific mechanism of circCCDC66 regulating tumorigenesis are required to provide new insights into the development of new biomarkers and personalized cancer treatments.

## Author contributions

XW and CZ drafted and revised the manuscript. HS and JY collected relevant papers and helped to revise the manuscript. LZ and JH reviewed the article. XW designed tables and charts. All authors contributed to the article and approved the submitted version.

## Glossary

**Table:**

circRNAs	Circular RNAs
ncRNAs	non-coding RNAs
COVID-19	coronavirus 2019
pre-mRNAs	precursor RNAs
EMT	epithelial-mesenchymal transition
CRC	colorectal cancer
HCC	hepatocellular carcinoma
ceRNAs	competing endogenous RNAs
RBPs	RNA binding proteins
ecircRNAs	exonic circRNAs
eiciRNAs	exon-intron circRNAs
ciRNAs	intronic circRNAs
miRNAs	microRNAs
CCDC66	the coiled-coil domain containing 66
JBTS	Joubert syndrome
TC	Thyroid cancer
PDK4	pyruvate dehydrogenase kinase 4
PTC	papillary TC
LARP1	La-related protein 1
LC	Lung cancer
SCLC	small-cell lung carcinom
NSCLC	non-small cell lung carcinoma
LADC	lung adenocarcinoma;
LUSC	lung squamous cell carcinoma
LCLC	large cell lung carcinoma
KPNA4	Karyopherin subunit alpha 4
STAT3	signal transducer and activator of transcription 3
SRCIN1	SRC kinase signaling inhibitor 1
TKIs	tyrosine kinase inhibitors
EGFR	epidermal growth factor receptor
SAE1	SUMO-activating enzyme subunit 1
HGF	Hepatocyte growth factor
GC	Gastric cancer
LHX2	LIM-homeobox domain 2
CDDP	Cisplatin
qRT-PCR	quantitative reverse transcription polymerase chain reaction
BCL2	B-cell lymphoma-2
DHX9	DExH-box helicase 9
PI3KKs	phosphatidylinositol 3-kinase-related kinases
MDM2	mouse double minute 2
MDM4	mouse double minute 4
RCC	renal cell carcinoma
REXO1	RNA exonuclease 1 homolog
FOXM1	forkhead box M1
OS	Osteosarcoma
PTP1B	protein tyrosine phosphatase 1B
HSCR	Hirschsprung disease
OA	Osteoarthritis
SIRT3	Sirtuin 3
AAA	Abdominal aortic aneurysm
VSMCs	vascular smooth muscle cells
CSCs	Cancer stem cells
DFS	disease-free survival
